# Patient satisfaction towards pharmacists’ services in the community pharmacies in the Hail region

**DOI:** 10.1371/journal.pone.0354731

**Published:** 2026-07-30

**Authors:** Tareq Nafea Alharby, Muteb Alanazi, Mukhtar Ansari, Ali Felaih Almutairy, Osama Ayed Altamimi, Saud Talal Jaafari, Saleh Abdulwahab Alassaf, Meshari Khalid Altuwayhir, Faisal Nasser Almutiri, Fahad Saad Aljohani

**Affiliations:** 1 Department of Clinical Pharmacy, College of Pharmacy, University of Ha’il, Hail, Saudi Arabia; 2 Department of Pharmacology and Toxicology, College of Pharmacy, Qassim University, Buraydah, Saudi Arabia; 3 College of Pharmacy, University of Ha’il, Hail, Saudi Arabia; University of Science and Technology of Fujairah, YEMEN

## Abstract

**Objectives:**

Research on patient satisfaction with community pharmacy services in Saudi Arabia has mostly concentrated on large cities, leaving a significant gap in understanding these services within the Hail region. Evaluating patient satisfaction in Hail is essential to address regional service gaps in privacy and counseling. Thus, this study was designed to assess the patient satisfaction with community pharmacy services in the Hail region.

**Methods:**

This cross-sectional study targeting the community pharmacy visitors in Hail region was conducted among 200 participants for a duration of three months (i.e., from 14 June 2024–29 September 2024). Patient satisfaction was assessed by a reliable and valid questionnaire. The instrument consisted of 17 items; and all of them were 5-point Likert scale responses (Poor = 1, Fair = 2, Good = 3, Very good = 4, Excellent = 5). To guarantee diverse geographic representation, 20 community pharmacies were purposefully chosen from several Hail City districts. In order to achieve balanced sample across all sites, a total of 200 participants were recruited, with roughly 10 patients joined from each Pharmacy. The data was analyzed for descriptive statistics using the SPSS, version 29.0.

**Results:**

The majority of the 200 participants in this study (mean age of 37.5 years) were married (60.5%) and male (56.5%). More than half had only completed high school, and only 16% had insurance.

Most of the patients (53%) consistently visited the same pharmacy. Pharmacist-patient relationships received the highest level of satisfaction, but accessibility—specifically, the availability of private consultation areas—received the lowest. Regular pharmacy visitors reported higher levels of satisfaction across multiple domains, whereas younger, male, single, better-educated, insured, and healthier patients had more favorable perceptions of medicine availability, highlighting disparities in demographic and health factors.

**Conclusion:**

The majority of community pharmacy visitors were young adults, married, had a range of educational backgrounds, and frequently used the services without a prescription or health insurance. Overall, patient satisfaction was good; nevertheless, to further improve patient experience and access to pharmacy services, improvements in private consultation facilities and pharmaceutical pricing are required.

## Introduction

Patients’ satisfaction with community pharmacy services is a crucial aspect of evaluating the quality and effectiveness of healthcare services provided. Community pharmacies play an important role in providing comprehensive pharmaceutical care delivery, offering services beyond dispensing medications, such as counseling [1]. Patient medication counselling is defined as “providing medication information orally or in written form to the patients or their representatives or providing proper directions of use, advice on side effects, storage, and diet and lifestyle modifications” [2].

Pharmacists play a crucial role in counseling patients regarding their medications and overall health [3]. As medication experts, pharmacists possess extensive knowledge about drugs, their uses, potential side effects, and interactions. This expertise enables pharmacists to provide valuable counseling and guidance to patients, ensuring safe and effective medication use. In addition to medication counseling, pharmacists also offer advice on general health and wellness, empowering patients to make informed decisions about their healthcare [4].

patient satisfaction is a multidimensional concept affected by the quality of care, accessibility, patient-pharmacist relationship and communication [5]. Patient satisfaction in the context of community pharmacy services is correlated with the efficacy of pharmacists’ counseling services. Recently, studies on patient satisfaction with community pharmacy services have drawn attention and most of them were conducted mostly in big cities such as Riyadh, Medina, Qassim and Makkah [6–9].

Although researches has been conducted on pharmacy services in major Saudi cities, the results cannot be applied to peripheral provinces like Hail, which have different infrastructure, economic, and geographic characteristics. Previous urban research ignores regional areas where an overwhelming 84% of the population lacks health insurance and relies on community pharmacies as a primary point of care for healthcare. Instead, it assumes a typical framework of insured consumers and organized clinical facilities. By carrying out a targeted assessment in the Hail region to comprehend how these local systemic limitations affect patient satisfaction and service accessibility, our study fills this crucial gap. Hence, evaluating patient satisfaction in Hail is essential to address regional service gaps in privacy and counseling and it plays a key role in identifying the areas for improvement including patient experiences. Thus, this study was designed with the aim to assess the patient satisfaction with community pharmacy services in the Hail region, providing a regional guide to improve clinical privacy laws, price equity, and infrastructure design outside of major urban centers.

## Materials and methods

### Study design

This was a quantitative – cross-sectional descriptive study targeting the community pharmacy visitors. Participants were included in the study if they aged 18 years old or more, live in Hail region, and able to understand and provide written informed consent. Any participants below 18 years of the age and living in other part of the country than Hail region, and not willing to participate in the study were excluded. This study was approved by the Research and Ethics Committee at the University of Ha’il, Saudi Arabia with approval number: H-2024–296

### Study duration and location

The data was gathered in the community pharmacies from the visitors in the Hail region for a duration of three months, i.e., from 14 June 2024–29 September 2024.

### Study setting, sample size and sampling procedure

The study's minimum required sample size was 384, calculated using 95% confidence level, a 5% margin of error, and an assumed response distribution of 50%. The 50% value was used solely as a statistical assumption for sample size estimation and does not represent the actual participant response rate in the study.

A total of 20 community pharmacies were included in this study. These pharmacies were purposively selected from different geographical districts across Hail city (e.g., Al-Aziziyah, Al-Wusayta, Al-Muhammadiyah, etc.) to ensure a diverse representation of pharmacy visitors. Regarding the distribution of subjects, we recruited a total of 200 participants, with a target of 10 patients per pharmacy (20 pharmacies × 10 patients = 200). This balanced approach allowed us to obtain a consistent and representative sample across all included sites.

In order to reduce selection bias and guarantee a diverse demographic mix, interviewers approached the pharmacy visitor leaving the premises during peak working hours. Researchers used a face-to-face survey methodology, where interviewers went directly to pharmacy visitors to explain the study and obtain their prompt consent, to reduce high non-response and dropout rates. However, despite these aggressive measures to increase engagement and guarantee eligibility through on-site assessments, the final sample only included 200 participants, short of the original goal of 384.

Data collectors were given standardized training to guarantee impartial and consistent questionnaire administration to reduce any bias. In order to minimize social desirability bias and promote truthful answers, interviews were held in a quiet location and participants were guaranteed anonymity. To guarantee language clarity and reduce response errors, the survey was also pre-tested and validated.

### Study tool

Patient satisfaction with pharmacists’ services was measured by a validated scale consisting of 17 items, developed by Hasan et al [10]. The responses of items were recorded, combined, and weighted to generate four scales of relationship, information, availability, and accessibility, having reliabilities, as measured by Cronbach's alpha, was of 0.92, 0.90, 0.74, and 0.87, respectively. The relationship scale consists of six items, while information scale with four items, availability scale with two items, and the accessibility scale with five items. All items in the survey were a 5-point Likert scale response (Poor = 1, Fair = 2, Good = 3, Very good = 4, Excellent = 5). The instrument also contained some questions related to sociodemographic characteristics of the participant, and health status.

### Data collection

Our study employed a face-to-face survey instrument designed to capture data on all three key variables of interest. The survey was administered in community pharmacies across the Hail region. Interviewers approached potential participants in the pharmacies. To ensure eligibility, interviewers utilized a brief screening questionnaire aligned with the study's inclusion and exclusion criteria. Interviewers provided a clear and concise explanation of the study's purpose and procedures. Participants provided written informed consent ensuring their voluntary participation and understanding of their rights. Upon obtaining informed consent, interviewers proceeded with the survey questions. All responses were recorded for further analysis.

### Data analysis

Means and standard deviations were used for continuous variables, while frequencies and percentages were used for categorical variables. The data was analyzed using the most up-to-date version of SPSS, version 29.0 to assess patient satisfaction towards pharmacists’ services in community pharmacies.

## Results

S1 Table summarizes the sociodemographic and health characteristics of the participants. The mean age of the participants was 37.48 years, and the most participants belonged to the 30–39 age group. More than half (56.5%) of respondents were men, and 60.5% were married. There was almost an even split in the education level of the participants: 51.5% had a high school education or less, and 48.5% had a college degree or more. The majority (84%) of respondents did not have health insurance coverage, while a little more than half (53%) reported that they consistently visited the same pharmacy. About half of the respondents (49%) reported having a long-term illness, and 43% were taking three or more medications. At the same time, 51% of participants said they did not take any prescription drugs, suggesting that over half of the cohort used community pharmacies mainly to get over-the-counter (OTC) pharmaceuticals, health supplements, or direct non-prescription clinical advice.

S2 Table shows the average scores of patient satisfaction towards the quality of community pharmacy services in four domains. The “Relationship” domain had the highest mean score (M = 3.74, SD = 0.99), and the “Accessibility” domain had the lowest mean score (M = 3.09, SD = 0.98). The question about the pharmacy's location got the highest score (M = 4.47, SD = 0.87) in the “Accessibility” category, while the question about the availability of a private consultation area received the lowest score (M = 2.07, SD = 1.43). In the “Information” category, tips on how to deal with common health problems achieved a high score (M = 3.85, SD = 1.23), while information about the side effects of medications showed a lower score (M = 2.89, SD = 1.50).

A one-way analysis of variance (ANOVA) was conducted to assess the association between the pharmacy service satisfaction and sociodemographic and health variables. S3 Table highlights four dimensions of patient satisfaction towards pharmacists’ services: relationship, information, availability, and accessibility, along with their corresponding mean scores and p-values for different sociodemographic and health subgroups. For the relationship, information, availability and accessibility, only one health variable (same pharmacy visit) was significantly associated at p = 0.01, p = 0.01, p = 0.04, p = 0.04 respectively. This finding indicates that patients who regularly visited the same pharmacy experienced a stronger relationship with the pharmacist, received more information and counseling, and had better availability of their medications and accessibility to services compared to patients who went to other pharmacies. This suggests that long-term familiarity between patients and pharmacists promotes a continuous care approach, reducing barriers to communication and maximizing transactional efficiency.

In contrast, medication availability was statistically significant with all socio-demographics and health variables (age, gender, relationship status, education level, health insurance, same pharmacy visit, chronic illness, prescribed medication) at *p* ≤ 0.05. Older participants had significantly lower availability scores compared to younger age groups. Patients taking more than two medications reported the lowest availability scores. This discrepancy draws attention to a systemic supply-chain vulnerability with relation to specialized treatments for chronic illnesses in remote areas. While younger cohorts have easy access to well-stocked, fast-moving over-the-counter items, older, multi-morbid patients’ complex multi-drug regimens are likely to experience local stock outs, requiring these vulnerable populations to navigate multiple facilities in order to meet their clinical needs. The medication availability decreased significantly across age groups, from younger to older groups. Similarly, younger aged males, single individuals, those with a college education and visiting same pharmacy, and patients without health insurance or a chronic illness, and those without any prescribed medication all reported significantly higher mean scores. This pattern suggests that younger, highly educated, and socio-demographically mobile people are better able to access and obtain essential health services than less mobile demographic groups because they have higher healthcare literacy and greater physical mobility. Patients visiting the same pharmacy reported higher mean scores in the availability of medications than those who visited various pharmacies

## Discussion

The main aim of this study was to evaluate patient satisfaction with community pharmacy services within the Hail region. The results offer important insights into patient demographics, medication behaviors, and perceptions of pharmacy care.

### Demographic profile and patient characteristics

Most participants were young adults, with a predominant proportion being married (60.5%) and showing a closely balanced distribution between high school (51.5%) and college-level education (48.5%). Males slightly outnumbered females among pharmacy visitors (56.5%), which is consistent with previous research showing gender differences in healthcare utilization. A significant portion of respondents (84%) reported not having health insurance, potentially affecting their dependence on community pharmacies for medication access and advice. Uninsured individuals may be more inclined to visit a pharmacy because they often depend on pharmacies for a variety of health-related needs, including over-the-counter medicines, basic health guidance, and medical supplies, instead of going to a healthcare provider. Pharmacies are easily accessible and convenient, making them a practical option for those without insurance, as they usually do not require appointments and can be more cost-effective [11,12]. Moreover, uninsured people might visit pharmacies more often to address minor health concerns or buy medications they cannot afford through other medical channels. Contradictly, a study by Alotaibi et al in Saudi Arabia found that participants who had health insurance were also more likely to visit a pharmacy [13]. This might be due to the reason that health insurance plans include coverage for community pharmacy visits, making it easier and more affordable for insured individuals to utilize pharmacy services regularly and conveniently [14]. Internationally, in structured referral systems like the UK’s National Health Service (NHS) or heavily insured European frameworks, pharmacy visits are tightly coupled with formal physician prescriptions [15,16]. Our results, however, point to a unique regional model in which community pharmacists serve as crucial safety point for primary care. The majority did not have chronic health conditions (87.5%), indicating that pharmacy visits were primarily for minor or acute health issues, aligning with the high frequency of consultations for common ailments.

### Medication use and chronic conditions

Among those with chronic illnesses, diabetes and hypertension were most common, reflecting non-communicable diseases in the region to be prevalent. The fact that more than two-thirds (67.5%) of patients obtained medications without prescriptions suggests that pharmacy services are easily accessible and may promote self-medication or immediate over-the-counter drug purchases. Studies have pointed towards prevalence of self-medication in the region [17–19]

### Patient satisfaction with pharmacy services

Overall, perceptions about pharmacy services were largely positive, with most respondents expressing satisfaction with the explanations given about medications, advice on managing common illnesses, and the professionalism of pharmacy staffs. Specifically, over 60% rated staff explanations, management advice, and staff expertise as excellent or very good. The findings of a study conducted in Jordan go in line with our study’s findings [20]. Another study conducted by Al-Arifi [8] found higher level (91.5%) of respondents’ satisfaction with the services provided by pharmacists. These findings indicate that community pharmacists are viewed as capable and reliable sources of medication-related information. A community pharmacy is a vital part of the healthcare delivery system, and they offer a wide range of services such as counseling, disease prevention and medication management. The level of satisfaction that patients have with the services of a community pharmacy is a vital indicator of the quality of their healthcare [9,21]. Nevertheless, some areas showed dissatisfaction such as the availability of private consultation spaces was notably rated poorly, with 57% of participants considering this aspect inadequate. Privacy is essential for effective counseling, especially when discussing sensitive health issues and the lack of private areas might hinder thorough and confidential discussions. Furthermore, more privacy is necessary for effective patient counseling in culturally particular contexts like Saudi Arabia. The findings of a study by Alrasheed et al also align with the findings of this study [22] Another study from Australia also highlights the challenges with private consultation space in community pharmacies [23] Although the physical layout of community pharmacies is a global privacy challenge, Saudi Arabia's cultural factor is sensibly linked with this. For normal counseling, a semi-private counter could be adequate in Western models; but, Saudi Arabia's distinct cultural values on gender privacy and family confidentiality require fully segregated, private spaces for patient engagement. Our poor privacy satisfaction scores (M = 2.07) clearly show that the physical infrastructure in outlying areas like Hail has fallen behind as the regulatory mandate turns toward increasing the clinical role of pharmacists, creating a bottleneck for efficient, private patient care. Additionally, about one-half of the participants expressed dissatisfaction with medication prices, possibly due to concerns over affordability or transparency. High medication prices pose a barrier to effective healthcare delivery, potentially leading to poorer health outcomes and increased societal costs. A global study on medicine prices, availability and affordability in 54 low- and middle-income countries also highlighted that high price of medicines will lead to poor health outcomes [24]. A scoping review in Saudi Arabia also highlighted the issue of price of medicine [24].

### Implications and suggestions

The high satisfaction levels with most pharmacy services demonstrate that pharmacists are effectively performing their pharmacy services in the region. However, the identified shortcomings—particularly regarding privacy and medication costs—warrant attention. Improving privacy by creating dedicated consultation areas could enable more comprehensive and confidential counseling, and ultimately enhancing patient trust. Addressing concerns related to medication pricing, perhaps through increased transparency or cost-control strategies, could further improve patient experiences.

Considering the heavy reliance on pharmacies for medication without prescriptions and the lack of insurance coverage for most patients, pharmacists could expand their role in patient education and health promotion. This would include emphasizing the safe use of medications and raising awareness about available health insurance options. Furthermore, policymakers could enforce private consultation areas through updated license criteria and introduce pricing transparency measures to address economic concerns among the uninsured in order to better align pharmacy services with public needs. Formalizing ‘Minor Ailment Schemes’ would also take use of the pharmacist's primary care function, and geriatric-specific treatments could address the reduced availability and satisfaction that older patients have expressed. A more patient-centered, fair healthcare delivery system throughout the Hail region would be ensured by these specific infrastructure and economic changes.

## Limitations

This study has some limitations, including its cross-sectional design and dependence on self-reported data, which may introduce bias. The study only obtained 200 completed questionnaires, falling short of the estimated sample size of 384 people. This smaller sample size might have affected the statistical capacity to identify weaker sociodemographic relationships and raised the margin of error. Additionally, the results might not accurately reflect the experiences of patients in other parts of Saudi Arabia because the sample was limited to this small area and depended on self-reported data. As a result, even while these insights are essential for regional advancements, care should be used when extrapolating these particular findings to larger national healthcare contexts.

Future research could incorporate qualitative methods to gain deeper insights into patient perceptions and assess the effectiveness of specific interventions aimed at enhancing pharmacy services.

## Conclusions

This study offers baseline information on patient satisfaction with community pharmacy services in the Hail region. The results indicate that community pharmacies are often used as point of contact for primary care by pharmacy visitors in this regional cohort, who are primarily young, married individuals. Significant structural deficiencies were found with regard to consultation privacy and the transmission of specialized health information, even though participants reported positive experiences with pharmacist interactions and general medication availability. These findings may not be extrapolated to Saudi Arabia's larger healthcare system due to the study's localized cross-sectional methodology and small sample size. In order to optimize pharmacy licensing infrastructures, local healthcare administrators could use these regional criteria as an exploratory framework. Future large-scale, multi-center longitudinal studies are needed to conclusively identify service satisfaction patterns across larger demographic and geographic areas.

## Supporting information

S1 Table Characteristics of study participants based on sociodemographic and health variables. (DOCX)

S2 Table Mean scores for patient evaluations of pharmacy service quality by domain. (DOCX)

S3 Table Mean for patient evaluations of pharmacy service quality by study population characteristics. (DOCX)

S1 Checklist STROBE checklist. (DOC)
