# Supplementary material for: Patient satisfaction towards pharmacists’ services in the community pharmacies in the Hail region
Source: PLoS One. 2026 Jul 30;21(7):e0354731. doi: 10.1371/journal.pone.0354731 (PMC13423062; doi:10.1371/journal.pone.0354731)
Supplement: S1 Table — (DOCX) [file pone.0354731.s001.docx]

| **Table 1. Characteristics of study participants based on sociodemographic and health variables** | | | |
| --- | --- | --- | --- |
| **Sociodemographic variables** | | **Frequency** | **Percent (%)** |
| **Age** M (SD) | | 37.48 (9.95) |  |
|  | 23-29 | 54 | 27 |
|  | 30-39 | 68 | 34 |
|  | 40-49 | 47 | 23.5 |
|  | 50 and more | 31 | 15.5 |
| **Gender** | |  |  |
|  | Female | 87 | 43.5 |
|  | Male | 113 | 56.5 |
| **Relationship status** | |  |  |
|  | Single | 79 | 39.5 |
|  | Married | 121 | 60.5 |
| **Education** | |  |  |
|  | High school and less | 103 | 51.5 |
|  | College and more | 97 | 48.5 |
| **Health insurance** | |  |  |
|  | Yes | 32 | 16 |
|  | No | 168 | 84 |
| **Same pharmacy visit** | |  |  |
|  | Yes | 106 | 53 |
|  | No | 94 | 47 |
| **Chronic illness** | |  |  |
|  | Yes | 98 | 49 |
|  | No | 102 | 51 |
| **Prescribed medication** | |  |  |
|  | No medication | 102 | 51 |
|  | One or two | 12 | 6 |
|  | Three or more | 86 | 43 |
| *Note*. *N* = 200. *M* = Mean, *SD*= Standard Deviation | | | |
