# Supplementary material for: Patient satisfaction towards pharmacists’ services in the community pharmacies in the Hail region
Source: PLoS One. 2026 Jul 30;21(7):e0354731. doi: 10.1371/journal.pone.0354731 (PMC13423062; doi:10.1371/journal.pone.0354731)
Supplement: S2 Table — (DOCX) [file pone.0354731.s002.docx]

| **Table ‎2. Mean scores for patient evaluations of pharmacy service quality by domain** | | |
| --- | --- | --- |
| **Characteristics** | | **Mean (*SD*)** |
| **Relationship** | | **3.74 (0.99)** |
|  | The explanation I receive from the pharmacy staff about my medications | 3.81 (1.20) |
|  | The interest shown by the pharmacy staff to help me make the best use of my medications | 3.57 (1.32) |
|  | The time that the pharmacy staff dedicates for attending to my needs | 3.69 (1.20) |
|  | My confidence in the expertise of the pharmacy staff | 3.89 (1.12) |
|  | My satisfaction with the overall pharmacy services | 3.71 (1.09) |
|  | My trust in the information provided by the pharmacy staff | 3.82 (1.17) |
| **Information** | | **3.28 (1.20)** |
|  | The information provided by the pharmacy staff about possible side effects of medications | 2.89 (1.50) |
|  | The advice provided on how to manage common ailments (e.g. colds &flu, fever, diarrhea) | 3.85 (1.23) |
|  | The advice provided on how to maintain a healthy lifestyle | 3 (1.48) |
|  | My overall satisfaction with the quality of information I receive at my usual pharmacy | 3.4 (1.31) |
| **Availability** | | **3.68 (1.13)** |
|  | The availability of prescription medications in my pharmacy | 3.52 (1.35) |
|  | The availability of medications that I buy without a prescription in my pharmacy | 3.85 (1.19) |
| **Accessibility** | | **3.09 (0.98)** |
|  | The location of my pharmacy | 4.47 (0.87) |
|  | The availability of a waiting area in my pharmacy | 3.08 (1.57) |
|  | The availability of private consultation (explanation) area in my pharmacy | 2.07 (1.43) |
|  | My satisfaction with medication prices | 2.76 (1.34) |
|  | My satisfaction with the overall insurance medication coverage (if available) is | 3.81 (1.20) |
| *Note*. *N =200*. | | |
