# Supplementary material for: Patient satisfaction towards pharmacists’ services in the community pharmacies in the Hail region
Source: PLoS One. 2026 Jul 30;21(7):e0354731. doi: 10.1371/journal.pone.0354731 (PMC13423062; doi:10.1371/journal.pone.0354731)
Supplement: S3 Table — (DOCX) [file pone.0354731.s003.docx]

| **Table 3. Mean for patient evaluations of pharmacy service quality by study population characteristics** | | | | | | | | | |
| --- | --- | --- | --- | --- | --- | --- | --- | --- | --- |
| **Characteristics** | | **Relationship** | | **Information** | | **Availability** | | **Accessibility** | |
|  |  | Mean | p-value* | Mean | p-value* | Mean | p-value* | Mean | p-value* |
| **Age** | |  | 0.78 |  | 0.32 |  | 0.001 |  | 0.33 |
|  | 23-29 | 3.82 |  | 3.44 |  | 4.28 |  | 3.20 |  |
|  | 30-39 | 3.68 |  | 3.21 |  | 4.05 |  | 3.16 |  |
|  | 40-49 | 3.68 |  | 3.07 |  | 3.07 |  | 2.87 |  |
|  | 50 and more | 3.85 |  | 3.48 |  | 2.75 |  | 3.07 |  |
| **Gender** | |  | 0.08 |  | 0.76 |  | 0.005 |  | 0.89 |
|  | Female | 3.89 |  | 3.31 |  | 3.43 |  | 3.08 |  |
|  | Male | 3.63 |  | 3.26 |  | 3.87 |  | 3.10 |  |
| **Relationship status** | |  | 0.42 |  | 0.09 |  | 0.01 |  | 0.09 |
|  | Single | 3.81 |  | 3.46 |  | 3.93 |  | 3.24 |  |
|  | Married | 3.69 |  | 3.16 |  | 3.51 |  | 3.00 |  |
| **Education** | |  | 0.83 |  | 0.64 |  | 0.001 |  | 0.53 |
|  | High school and less | 3.76 |  | 3.25 |  | 3.32 |  | 3.05 |  |
|  | College and more | 3.73 |  | 3.32 |  | 4.10 |  | 3.14 |  |
| **Health insurance** | |  | 0.26 |  | 0.34 |  | 0.003 |  | 0.33 |
|  | Yes | 3.93 |  | 3.47 |  | 3.14 |  | 2.94 |  |
|  | No | 3.71 |  | 3.25 |  | 3.79 |  | 3.12 |  |
| **Same pharmacy visit** | |  | 0.01 |  | 0.01 |  | 0.04 |  | 0.04 |
|  | Yes | 3.91 |  | 3.49 |  | 3.84 |  | 3.23 |  |
|  | No | 3.56 |  | 3.05 |  | 3.51 |  | 2.94 |  |
| **Chronic illness** | |  | 0.63 |  | 0.30 |  | 0.001 |  | 0.07 |
|  | Yes | 3.71 |  | 3.19 |  | 3.14 |  | 2.96 |  |
|  | No | 3.78 |  | 3.37 |  | 4.20 |  | 3.21 |  |
| **Prescribed medication** | |  | 0.23 |  | 0.08 |  | 0.001 |  | 0.20 |
|  | No medication | 3.78 |  | 3.37 |  | 4.20 |  | 3.21 |  |
|  | One or two | 3.26 |  | 2.54 |  | 3.79 |  | 2.92 |  |
|  | Three or more | 3.77 |  | 3.28 |  | 3.05 |  | 2.97 |  |
| *Note*. *N* = 200. *M* = Mean, *SD*= Standard Deviation | | | | | | | | | |
