# Supplementary material for: Patient satisfaction towards pharmacists’ services in the community pharmacies in the Hail region
Source: PLoS One. 2026 Jul 30;21(7):e0354731. doi: 10.1371/journal.pone.0354731 (PMC13423062; doi:10.1371/journal.pone.0354731)
Supplement: Attachment — Submitted filename: PONE-D-25-61986_reviewer.pdf [file pone.0354731.s005.pdf]

PLOS ONE

Patient satisfaction towards pharmacists’ services in the community pharmacies in the Hail region

--Manuscript Draft--

**Manuscript Number:**	PONE-D-25-61986
**Article Type:**	Research Article
**Full Title:**	Patient satisfaction towards pharmacists’ services in the community pharmacies in the Hail region
**Short Title:**	Patient satisfaction in community pharmacies
**Corresponding Author:**	Mukhtar Ansari, Ph D<br>University of Ha'il<br>Hail, Hail SAUDI ARABIA
**Keywords:**	Adult; patient satisfaction; Community pharmacy; Hail region; services; Saudi Arabia
**Abstract:**	Objectives<br>To assess the patient satisfaction with community pharmacy services in Hail region.<br>Methods<br>This is a quantitative - cross-sectional descriptive study targeting the community pharmacy visitors in Hail region for a duration of three months (i.e., from 14 June 2024 to 29 September 2024) among 200 participants. Patient satisfaction was measured by a reliable and valid questionnaire that was developed by Hasan et al. The instrument has 17 items; all of them are 5-point Likert scale responses (Poor= 1, Fair = 2, Good = 3, Very good = 4, Excellent = 5) The data was analyzed for descriptive statistics using the SPSS, version 29.0.<br>Results<br>This study of 200 participants (mean age 37.5 years) found most were male (56.5%) and married (60.5%), with over half having high school education or less and only 16% insured. Most patients (53%) consistently visited the same pharmacy. Satisfaction was highest in the relationship domain, especially confidence in staff, and lowest in accessibility, especially private consultation areas. Regular pharmacy visitors reported higher satisfaction across multiple domains. Medication availability was significantly linked to sociodemographics; younger, male, single, higher-educated, insured, and healthier patients reported better availability. Availability decreased with age and medication burden, revealing disparities based on demographic and health factors.<br>Conclusion<br>The study results indicate that young adults, who were mostly unmarried and well educated, frequently utilize community pharmacy services, mainly without prescription drugs or health insurance. Although there are still issues with private consultation rooms and medication costs, overall satisfaction with pharmacy services was high. These insights highlight the need for improvements in privacy facilities and transparent pricing to enhance patient experience and access to the pharmacy.
**Order of Authors:**	Tareq Nafea Alharby, PhD
	Muteb Alanazi, PhD
	Mukhtar Ansari, PhD
	Ali Felaih Almutairy, PhD
	Osama Ayed Altamimi, PharmD
	Saud Talal Jaafari, PharmD
	Saleh Abdulwahab Alassaf, PharmD
	Meshari Khalid Altuwayhir, PharmD
	Faisal Nasser Almutiri, PharmD
	Fahad Saad Aljohani, PharmD

Powered by Editorial Manager® and ProduXion Manager® from Aries Systems Corporation

Additional Information:	
**Question**	**Response**
**Financial Disclosure**<br><br>Enter a financial disclosure statement that describes the sources of funding for the work included in this submission. Review the submission guidelines for detailed requirements. View published research articles from *PLOS ONE* for specific examples.<br><br>This statement is required for submission and **will appear in the published article** if the submission is accepted. Please make sure it is accurate.<br><br>**Funded studies**<br>Enter a statement with the following details:<br>• Initials of the authors who received each award<br>• Grant numbers awarded to each author<br>• The full name of each funder<br>• URL of each funder website<br>• Did the sponsors or funders play any role in the study design, data collection and analysis, decision to publish, or preparation of the manuscript?<br><br>Did you receive funding for this work?	Yes
Please add funding details.<br>as follow-up to "**Financial Disclosure**<br><br>Enter a financial disclosure statement that describes the sources of funding for the work included in this submission. Review the submission guidelines for detailed requirements. View published research articles from *PLOS ONE* for specific examples.<br><br>This statement is required for submission and **will appear in the published article** if the submission is accepted. Please make sure it is accurate.	This research has been funded by Scientific Research Deanship at University of Ha'il - Saudi Arabia through project number RG-23 038.

**Funded studies**<br>Enter a statement with the following details:<br>• Initials of the authors who received each award<br>• Grant numbers awarded to each author<br>• The full name of each funder<br>• URL of each funder website<br>• Did the sponsors or funders play any role in the study design, data collection and analysis, decision to publish, or preparation of the manuscript?<br><br>Did you receive funding for this work?"	
Please select the country of your main research funder (please select carefully as in some cases this is used in fee calculation).<br>as follow-up to "**Financial Disclosure**<br><br>Enter a financial disclosure statement that describes the sources of funding for the work included in this submission. Review the submission guidelines for detailed requirements. View published research articles from *PLOS ONE* for specific examples.<br><br>This statement is required for submission and **will appear in the published article** if the submission is accepted. Please make sure it is accurate.<br><br>**Funded studies**<br>Enter a statement with the following details:<br>• Initials of the authors who received each award<br>• Grant numbers awarded to each author<br>• The full name of each funder<br>• URL of each funder website<br>• Did the sponsors or funders play any role in the study design, data collection and analysis, decision to publish, or preparation of the manuscript?<br><br>Did you receive funding for this work?"	SAUDI ARABIA - SA
**Competing Interests**	The authors have declared that no competing interests exist.

Use the instructions below to enter a competing interest statement for this submission. On behalf of all authors, disclose any competing interests that could be perceived to bias this work—acknowledging all financial support and any other relevant financial or non-financial competing interests.<br><br>This statement is required for submission and **will appear in the published article** if the submission is accepted. Please make sure it is accurate and that any funding sources listed in your Funding Information later in the submission form are also declared in your Financial Disclosure statement.<br><br>View published research articles from *PLOS ONE* for specific examples.<br><br>**NO authors have competing interests**<br><br>Enter: *The authors have declared that no competing interests exist.*<br><br>**Authors with competing interests**<br><br>Enter competing interest details beginning with this statement:<br><br>*I have read the journal's policy and the authors of this manuscript have the following competing interests: [insert competing interests here]*<br><br>* typeset	
**Ethics Statement**<br><br>Enter an ethics statement for this submission. This statement is required if the study involved:<br><br>• Human participants<br>• Human specimens or tissue<br>• Vertebrate animals or cephalopods<br>• Vertebrate embryos or tissues	Research and Ethics Committee at the University of Ha'il, Saudi Arabia with approval number: H-2024-296

Powered by Editorial Manager® and ProduXion Manager® from Aries Systems Corporation

• Field research<br><br>Write "N/A" if the submission does not require an ethics statement.<br><br>General guidance is provided below. Consult the submission guidelines for detailed instructions. **Make sure that all information entered here is included in the Methods section of the manuscript.**	

Powered by Editorial Manager® and ProduXion Manager® from Aries Systems Corporation

**Format for specific study types**<br><br>**Human Subject Research (involving human participants and/or tissue)**<br>• Give the name of the institutional review board or ethics committee that approved the study<br>• Include the approval number and/or a statement indicating approval of this research<br>• Indicate the form of consent obtained (written/oral) or the reason that consent was not obtained (e.g. the data were analyzed anonymously)<br><br>**Animal Research (involving vertebrate animals, embryos or tissues)**<br>• Provide the name of the Institutional Animal Care and Use Committee (IACUC) or other relevant ethics board that reviewed the study protocol, and indicate whether they approved this research or granted a formal waiver of ethical approval<br>• Include an approval number if one was obtained<br>• If the study involved *non-human primates*, add *additional details* about animal welfare and steps taken to ameliorate suffering<br>• If anesthesia, euthanasia, or any kind of animal sacrifice is part of the study, include briefly which substances and/or methods were applied<br><br>**Field Research**<br><br>Include the following details if this study involves the collection of plant, animal, or other materials from a natural setting:<br>• Field permit number<br>• Name of the institution or relevant body that granted permission	
**Data Availability**<br><br>Authors are required to make all data underlying the findings described fully available, without restriction, and from the time of publication. PLOS allows rare exceptions to address legal and ethical concerns. See the PLOS Data Policy and FAQ for detailed information.	Yes - all data are fully available without restriction

Powered by Editorial Manager® and ProduXion Manager® from Aries Systems Corporation

A Data Availability Statement describing where the data can be found is required at submission. Your answers to this question constitute the Data Availability Statement and **will be published in the article**, if accepted.<br><br>**Important:** Stating 'data available on request from the author' is not sufficient. If your data are only available upon request, select 'No' for the first question and explain your exceptional situation in the text box.<br><br>Do the authors confirm that all data underlying the findings described in their manuscript are fully available without restriction?	
**Describe where the data may be found in full sentences. If you are copying our sample text, replace any instances of XXX with the appropriate details.**<br><br>• If the data are **held or will be held in a public repository**, include URLs, accession numbers or DOIs. If this information will only be available after acceptance, indicate this by ticking the box below. For example: *All XXX files are available from the XXX database (accession number(s) XXX, XXX.)*.<br>• If the data are all contained **within the manuscript and/or Supporting Information files**, enter the following: *All relevant data are within the manuscript and its Supporting Information files.*<br>• If neither of these applies but you are able to provide **details of access elsewhere**, with or without limitations, please do so. For example:<br><br>*Data cannot be shared publicly because of [XXX]. Data are available from the XXX Institutional Data Access / Ethics Committee (contact via XXX) for researchers who meet the criteria for access to confidential data.*<br><br>*The data underlying the results presented in the study are available from (include the name of the third party*	The data underlying the results presented in the study are available from (the corresponding author at: mukhtaransari@hotmail.com

and contact information or URL).<br>• This text is appropriate if the data are owned by a third party and authors do not have permission to share the data.<br><br>* typeset	
Additional data availability information:	

Powered by Editorial Manager® and ProduXion Manager® from Aries Systems Corporation

Manuscript Click here to access/download;Manuscript;Manuscript File.docx

Patient satisfaction towards pharmacists' services in the community pharmacies in the Hail region

Tareq Nafea Alharby[1], Muteb Alanazi[1], Mukhtar Ansari[1*], Ali Felaih Almutairy[2], Osama Ayed Altamimi[3], Saud Talal Jaafari[3], Saleh Abdulwahab Alassaf[3], Meshari Khalid Altuwayhir[3], Faisal Nasser Almutiri[3], Fahad Saad Aljohani[3]

[1]Department of Clinical Pharmacy, College of Pharmacy, University of Ha'il, Hail 81442, Saudi Arabia

[2]Department of Pharmacology and Toxicology, College of Pharmacy, Qassim University, Buraydah 52571, Saudi Arabia

[3]College of Pharmacy, University of Ha'il, Hail 81442, Saudi Arabia

[*]Corresponding author: Mukhtar Ansari, Department of Clinical Pharmacy, College of Pharmacy, University of Ha'il, Hail 81442, Saudi Arabia, E-mail: mukhtaransari@hotmail.com

Short title: Patient satisfaction in community pharmacies

Abstract

Objectives

To assess the patient satisfaction with community pharmacy services in Hail region.

Methods

This is a quantitative - cross-sectional descriptive study targeting the community pharmacy visitors in Hail region for a duration of three months (i.e., from 14 June 2024 to 29 September 2024) among 200 participants. Patient satisfaction was measured by a reliable and valid questionnaire that was developed by Hasan et al. The instrument has 17 items; all of them are 5-point Likert scale responses (Poor= 1, Fair = 2, Good = 3, Very good = 4, Excellent = 5) The data was analyzed for descriptive statistics using the SPSS, version 29.0.

Results

This study of 200 participants (mean age 37.5 years) found most were male (56.5%) and married (60.5%), with over half having high school education or less and only 16% insured. Most patients (53%) consistently visited the same pharmacy. Satisfaction was highest in the relationship domain, especially confidence in staff, and lowest in accessibility, especially private consultation areas. Regular pharmacy visitors reported higher satisfaction across multiple domains. Medication availability was significantly linked to sociodemographics; younger, male, single, higher-educated, insured, and healthier patients reported better availability. Availability decreased with age and medication burden, revealing disparities based on demographic and health factors.

Conclusion

The study results indicate that young adults, who were mostly unmarried and well educated, frequently utilize community pharmacy services, mainly without prescription drugs or health

insurance. Although there are still issues with private consultation rooms and medication costs, overall satisfaction with pharmacy services was high. These insights highlight the need for improvements in privacy facilities and transparent pricing to enhance patient experience and access to the pharmacy.

Keywords: Adult, patient satisfaction, Community pharmacy, Hail region, services, Saudi Arabia

Introduction

Patients' satisfaction with community pharmacy services is a crucial aspect of evaluating the quality and effectiveness of healthcare services provided. Community pharmacies play an important role in providing comprehensive pharmaceutical care delivery, offering services beyond dispensing medications, such as counseling (1). Patient medication counselling defined as “providing medication information orally or in written form to the patients or their representatives or providing proper directions of use, advice on side effects, storage, and diet and lifestyle modifications” (2).

Pharmacists play a crucial role in counseling patients regarding their medications and overall health (3). As medication experts, pharmacists possess extensive knowledge about drugs, their uses, potential side effects, and interactions. This expertise enables pharmacists to provide valuable counseling and guidance to patients, ensuring safe and effective medication use. In addition to medication counseling, pharmacists also offer advice on general health and wellness, empowering patients to make informed decisions about their healthcare (4).

Patient satisfaction is a multidimensional concept affected by the quality of care, accessibility, and patient-pharmacist relationship and communication (5). In the realm of community pharmacy services, patient satisfaction is adhered to the counseling effectiveness offered by pharmacists. Recently, studies on patient satisfaction with community pharmacy services have drawn attention and most of them were conducted mostly in big cities such as Riyadh, Medina, Qassim and Makkah (6–9). Research on patient satisfaction with community pharmacy services in the Hail region is limited but essential for enhancing healthcare delivery.

Evaluating patient’s satisfaction with counseling services in community pharmacies, especially in Hail region, play a key role in identifying the areas for improvement including

patient experiences. Thus, this study was designed with the aim to assess the patient satisfaction with community pharmacy services in Hail regio

Materials and Methods

Study design

This was a quantitative - cross-sectional descriptive study targeting the community pharmacy visitors. Participants were included in the study if they aged 18 years old or more, live in Hail region, and able to understand and provide written informed consent. Any participants below 18 years of the age and living in other part of the country than Hail region, and not willing to participate were excluded from the study. This study was approved by the Research and Ethics Committee at the University of Ha'il, Saudi Arabia with approval number: H-2024-296

Study duration and location

The data was gathered in the community pharmacies from the visitors in the Hail region for a duration of three months, from 14 June 2024 to 29 September 2024.

Sample size and sampling procedure

The study's minimum effective sample size was 384, which was determined using a 50% response pattern, an error margin of 5%, and a 95% confidence level. However, due to high no response and drop out, the actual size of the sample was 200 who completed the questionnaire.

Study tool

Patient satisfaction with pharmacists' services was measured by a validated scale consisting of 17 items, developed by Hasan et al (10). The responses of items were recorded, combined, and weighted to generate four scales of relationship, information, availability, and accessibility, having reliabilities, as measured by Cronbach's alpha, was of 0.92, 0.90, 0.74, and 0.87, respectively. The relationship scale consists of six items, information scale four items, availability scale two items, and the accessibility scale five items. All items in the survey are a 5-point Likert scale response (Poor= 1, Fair = 2, Good = 3, Very good = 4, Excellent = 5). The instrument also contained some questions related to sociodemographic characteristics of the participant, and health status.

Data collection

Our study employed a face-to-face survey instrument designed to capture data on all three key variables of interest. The survey was administered in community pharmacies across the Hail region. Interviewers approached potential participants in the pharmacies. To ensure eligibility, interviewers utilized a brief screening questionnaire aligned with the study's inclusion and exclusion criteria. Interviewers provided a clear and concise explanation of the study's purpose and procedures. Participants provided written informed consent ensuring their voluntary participation and understanding of their rights. Upon obtaining informed consent, interviewers proceeded with the survey questions. All responses were recorded for further analysis.

Data analysis

Means and standard deviations were used for continuous variables, while frequencies and percentages were used for categorical variables. The data was analyzed using the most up-to-date version of SPSS, version 29.0 in order to assess patient satisfaction towards pharmacists' services in community pharmacies.

Results

Table 1 summarizes the sociodemographic and health characteristics of the 200 study participants. The mean age of the participants was 37.48 years, and the most participants belonged to the 30-39 age group. More than half (56.5%) of respondents were men, and nearly two-thirds (60.5%) were married. There was almost an even split in the education level of the participants: 51.5% had a high school education or less, and 48.5% had a college degree or more. The majority (84%) of respondents did not have health insurance coverage. A little more than half (53%) reported they consistently visited the same pharmacy. About half of the respondents (49%) reported having a long-term illness, and a large number (43%) said they were taking three or more medications.

Table 2 shows the average scores of patient satisfaction towards the quality of community pharmacy services in four domains. The "Relationship" domain had the highest mean score (M = 3.74, SD = 0.99), and the "Accessibility" domain had the lowest mean score (M = 3.09, SD = 0.98). The question about the pharmacy's location got the highest score (M = 4.47, SD = 0.87) in the "Accessibility" category, while the question about the availability of a private consultation area got the lowest score (M = 2.07, SD = 1.43). In the "Information" category, tips on how to deal with common health problems got a high score (M = 3.85, SD = 1.23), while information

about the side effects of medications got a lower score (M = 2.89, SD = 1.50).

A one-way analysis of variance (ANOVA) was conducted to assess the association between the pharmacy service satisfaction and sociodemographics and health variables. Table 3 highlights four dimensions of patient satisfaction towards pharmacists' services: relationship, information, availability, and accessibility, along with their corresponding mean scores and p-values for different sociodemographic and health subgroups. For the relationship, information, availability and accessibility, only one health variable (same pharmacy visit) was significant at p=0.01, p=0.01, p=0.04, p=0.04 respectively. This finding indicates that patients who regularly went to the same pharmacy experienced a stronger relationship with the pharmacist, received more information and counseling, and had better availability of their medications and accessibility to services compared to patients who went to other pharmacies.

In contrast, medication availability was statistically significant with all sociodemographics and health variables (age, gender, relationship status, education level, health insurance, same pharmacy visit, chronic illness, prescribed medication) at $p \leq 0.05$. Older participants had significantly lower availability scores compared to younger age groups. The medication availability decreased significantly across age groups, from younger to older groups. Similarly, younger aged males, single individuals, those with a college education and visiting same pharmacy, and patients without health insurance or a chronic illness, and those without any prescribed medication all reported significantly higher mean scores. Patients visiting the same pharmacy reported higher mean scores in the availability of medications than those who visiting

various pharmacies. Patients taking more than two medications reported the lowest availability scores.

Discussion

The main aim of this study was to evaluate patient satisfaction with community pharmacy services within the Hail region. The results offer important insights into patient demographics, medication behaviors, and perceptions of pharmacy care.

Demographic profile and patient characteristics

Most participants were young adults between 18 and 29 years old, mainly single and holding college-level education or higher. Males slightly outnumbered females among pharmacy visitors (56.5%), which is consistent with previous research showing gender differences in healthcare utilization. A significant portion of respondents (84%) reported not having health insurance, potentially affecting their dependence on community pharmacies for medication access and advice. Uninsured individuals may be more inclined to visit a pharmacy because they often depend on pharmacies for a variety of health-related needs, including over-the-counter medicines, basic health guidance, and medical supplies, instead of going to a healthcare provider. Pharmacies are easily accessible and convenient, making them a practical option for those without insurance, as they usually do not require appointments and can be more cost-effective. Moreover, uninsured people might visit pharmacies more often to address minor health concerns or buy medications they cannot afford through other medical channels. Contradictly, a study by Alotaibi et al in Saudi Arabia found that participants who had health insurance were also more likely to visit a pharmacy (11). This might be due to the reason that health insurance plans

include coverage for community pharmacy visits, making it easier and more affordable for insured individuals to utilize pharmacy services regularly and conveniently (12). The majority did not have chronic health conditions (87.5%), indicating that pharmacy visits were primarily for minor or acute health issues, aligning with the high frequency of consultations for common ailments.

Medication use and chronic conditions

Among those with chronic illnesses, diabetes and hypertension were most common, reflecting non-communicable diseases in the region to be prevalent. The fact that more than two-thirds (67.5%) of patients obtained medications without prescriptions suggests that pharmacy services are easily accessible and may promote self-medication or immediate over-the-counter drug purchases. Studies have pointed towards prevalence of self-medication in the region (13–15).

Patient satisfaction with pharmacy services

Overall, perceptions of pharmacy services were largely positive, with most respondents expressing satisfaction with the explanations given about medications, advice on managing common illnesses, and the professionalism of pharmacy staffs. Specifically, over 60% rated staff explanations, management advice, and staff expertise as excellent or very good. The findings of a study conducted in Jordan go in line with our study's findings (16). Another study conducted by Al-Arifi (8) found higher level (91.5%) of respondents' satisfaction with the services provided by pharmacists. These findings indicate that community pharmacists are viewed as capable and reliable sources of medication-related information. A community pharmacy is a vital part of the healthcare delivery system, and they offer a wide range of services such as

counseling, disease prevention and medication management. The level of satisfaction that patients have with the services of a community pharmacy is a vital indicator of the quality of their healthcare (9,17). Nevertheless, some areas showed dissatisfaction such as the availability of private consultation spaces was notably rated poorly, with 57% of participants considering this aspect inadequate. Privacy is essential for effective counseling, especially when discussing sensitive health issues and the lack of private areas might hinder thorough and confidential discussions. Furthermore, more privacy is necessary for effective patient counseling in culturally particular contexts like Saudi Arabia. The findings of a study by Alrasheed et al also align with the findings of this study (18). Another study from Australia also highlights the challenges with private consultation space in community pharmacies (19). Additionally, about one-half of the participants expressed dissatisfaction with medication prices, possibly due to concerns over affordability or transparency. High medication prices pose a barrier to effective healthcare delivery, potentially leading to poorer health outcomes and increased societal costs. A global study on medicine prices, availability and affordability in 54 low- and middle-income countries also highlighted that high price of medicines will lead to poor health outcomes (20). A scoping review in Saudi Arabia also highlighted the issue of price of medicine (20).

Implications and suggestions

The high satisfaction levels with most pharmacy services demonstrate that pharmacists are effectively performing their pharmacy services in the region. However, the identified shortcomings—particularly regarding privacy and medication costs—warrant attention. Improving privacy by creating dedicated consultation areas could enable more comprehensive and confidential counseling, and ultimately enhancing patient trust. Addressing concerns related

to medication pricing, perhaps through increased transparency or cost-control strategies, could further improve patient experiences.

Considering the heavy reliance on pharmacies for medication without prescriptions and the lack of insurance coverage for most patients, pharmacists could expand their role in patient education and health promotion. This would include emphasizing the safe use of medications and raising awareness about available health insurance options.

Limitations

This study has some limitations, including its cross-sectional design and dependence on self-reported data, which may introduce bias. Future research could incorporate qualitative methods to gain deeper insights into patient perceptions and assess the effectiveness of specific interventions aimed at enhancing pharmacy services.

Conclusions

Community pharmacies in the Hail region are generally well regarded by patients, especially regarding staffs' competence and the quality of medication information provided. To further improve patients' satisfaction and health outcomes, addressing issues related to privacy and medication affordability should be prioritized. Upgrading pharmacy infrastructure and service delivery models could promote a more patient-centered approach, ultimately supporting safer medication practices and improved health literacy within the community.

Supporting information

S1 Table. Characteristics of study participants based on sociodemographic and health variables.

S2 Table. Mean scores for patient evaluations of pharmacy service quality by domain.

S3 Table. Mean for patient evaluations of pharmacy service quality by study population characteristics.

S1 Checklist. STROBE checklist.

Author contributions

Conceptualization: TNA, MAlanazi, MAnsari, AFA, OAA, STJ, SAA, MKA, FNA, FSA

Formal analysis: TNA, MAlanazi, MAnsari, AFA

Funding acquisition: TNA

Methodology: TNA, MAlanazi, MAnsari, AFA, OAA, STJ, SAA, MKA, FNA, FSA

Project administration: TNA, MAlanazi, MAnsari

Supervision: TNA, MAlanazi, MAnsari

Writing – original draft: TNA, MAlanazi, MAnsari, AFA

Writing – review & editing: TNA, MAlanazi, MAnsari, AFA, OAA, STJ, SAA, MKA, FNA, FSA

Acknowledgment

This research has been funded by Scientific Research Deanship at University of Ha'il - Saudi Arabia through project number RG-23 038. However, the funder had no role in study design, data collection and analysis, decision to publish, or preparation of the manuscript.

Financial support

Nil

Conflicts of interest

None reported

References

1. Ford RC, Bach SA, Fottler MD. Methods of measuring patient satisfaction in health care organizations. Health Care Manage Rev. 1997;22(2):74–89.
2. Palaian S, Prabhu M, Shankar PR. Patient counseling by pharmacist-a focus on chronic illness. Pak J Pharm Sci. 2006;19(1):65–72.
3. Dalton K, Byrne S. Role of the pharmacist in reducing healthcare costs: current insights. Integr Pharm Res Pract. 2017;37–46.
4. Bell J, Dziekan G, Pollack C, Mahachai V. Self-care in the twenty first century: a vital role for the pharmacist. Adv Ther. 2016;33:1691–703.
5. AlGhurair SA, Simpson SH, Guirguis LM. What elements of the patient–pharmacist relationship are associated with patient satisfaction? Patient Prefer Adherence. 2012;663–76.
6. Alfadl AA, Alrasheedy AA, Alhassun MS. Evaluation of medication counseling practice at community pharmacies in Qassim region, Saudi Arabia. Saudi pharmaceutical journal. 2018;26(2):258–62.
7. Al-Tannir M, Alharbi AI, Alfawaz AS, Zahran RI, AlTannir M. Saudi adults satisfaction with community pharmacy services. Springerplus. 2016;5:1–5.

8. Al-Arifi MN. Patients' perception, views and satisfaction with pharmacists' role as health care provider in community pharmacy setting at Riyadh, Saudi Arabia. Saudi Pharmaceutical Journal. 2012;20(4):323–30.

9. El-Kholy AA, Abdelaal K, Alqhtani H, Abdel-Wahab BA, Abdel-Latif MMM. Publics' perceptions of community pharmacists and satisfaction with pharmacy services in Al-Madinah City, Saudi Arabia: a cross sectional study. Medicina (B Aires). 2022;58(3):432.

10. Hasan S, Sulieman H, Stewart K, Chapman CB, Hasan MY, Kong DCM. Assessing patient satisfaction with community pharmacy in the UAE using a newly-validated tool. Research in Social and Administrative Pharmacy. 2013;9(6):841–50.

11. Alotaibi MM, Almuharifi FY, Almuhaini DS, Alsulaiman DR, Albader MA, Alhejji WA, et al. Assessing Public Awareness, Utilization and Satisfaction with Community Pharmacy Services. Patient Prefer Adherence. 2024;1183–93.

12. Alomi YA. Community Pharmacy Policy and Procedures: Clinical Compounding Services. International Journal of Pharmacology and Clinical Sciences,. 2023;13(1).

13. Alhomoud F, Aljamea Z, Almahasnah R, Alkhalifah K, Basalelah L, Alhomoud FK. Self-medication and self-prescription with antibiotics in the Middle East—do they really happen? A systematic review of the prevalence, possible reasons, and outcomes. International journal of infectious diseases. 2017;57:3–12.

14. Alhur A, Alhur A, Alfayiz A, Alotaibi A, Hansh B, Ghasib N, et al. Patterns and prevalence of self-medication in Saudi Arabia: insights from a nationwide survey. Cureus. 2023;15(12).

15. Ansari M, Alanazi A, Moin A. Consumers' awareness, attitude and associated factors towards self-medication in Hail, Saudi Arabia. PLoS One. 2020;15(4):e0232322.

16. Naser AY, Sbeat BSA. Satisfaction with community pharmacies services in Jordan: A cross-sectional study. Saudi Pharmaceutical Journal. 2022;30(11):1646–51.

17. Bîrsan M, Focșa AV, Cristofor AC, Scripcariu SI, Antonoaea P, Vlad RA, et al. Healthcare service quality evaluated using the degree of satisfaction of patients in Romanian community pharmacies. In: Healthcare. MDPI; 2023. p. 3038.

18. Alrasheed MA, Alfageh BH, Almohammed OA. Privacy in Community Pharmacies in Saudi Arabia: A Cross-Sectional Study. In: Healthcare. 2024. p. 1740.

19. Hattingh HL, Emmerton L, Ng Cheong Tin P, Green C. Utilization of community pharmacy space to enhance privacy: a qualitative study. Health Expectations. 2016;19(5):1098–110.

20. Oldfield L, Penm J, Mirzaei A, Moles R. Prices, availability, and affordability of adult medicines in 54 low-income and middle-income countries: evidence based on a secondary analysis. Lancet Glob Health. 2025;13(1):e50–8.

Table 1

Click here to access/download
Supporting Information
S1 Table 1.docx

Table 2

Click here to access/download
Supporting Information
S1 Table 2.docx

Table 3

Click here to access/download
Supporting Information
S1 Table 3.docx

Strobe checklist

Click here to access/download
Supporting Information
S1 Checklist-STROBE Checklist.doc
