# Supplementary material for: Patient satisfaction towards pharmacists’ services in the community pharmacies in the Hail region
Source: PLoS One. 2026 Jul 30;21(7):e0354731. doi: 10.1371/journal.pone.0354731 (PMC13423062; doi:10.1371/journal.pone.0354731)
Supplement: Attachment — Submitted filename: Response to Reviewer 3.docx [file pone.0354731.s007.docx]

**Response to Reviewers Comments**

PONE-D-25-61986

Patient satisfaction towards pharmacists’ services in the community pharmacies in the Hail region

**Reviewer #3:**

The manuscript addresses an important topic—patient satisfaction with community pharmacy services in the Hail region. However, the novelty and scientific contribution of the study remain limited. Similar cross-sectional studies assessing patient satisfaction in community pharmacies have already been widely conducted in Saudi Arabia and internationally. The authors should clearly articulate what new knowledge this study adds beyond existing literature.

Clarification: We acknowledge the reviewer's comment regarding the prevalence of patient satisfaction studies. Although patient satisfaction studies are frequently conducted in large cities, this study is particularly valuable since it reveals unique infrastructure and socioeconomic issues unique to a non-metropolitan, peripheral location. By exposing a serious dearth of private consultation spaces (M = 2.07), a substantial reliance on pharmacies by uninsured individuals (84%), and notable declines in drug availability among older, multimedicated patients, it offers a crucial empirical roadmap for area policymakers.

The introduction is overly descriptive and lacks a clearly defined research gap. While several previous Saudi studies are cited, the authors do not critically compare those findings nor explain how the current study advances the field. The rationale for focusing specifically on the Hail region needs stronger justification.

Clarification: We appreciate the reviewer's constructive critique regarding the introduction's lack of a critical research gap. We have explained the justification in details in introduction section.

The sampling procedure raises methodological concerns. Although the authors calculated a required sample size of 384, the final sample included only 200 participants. This substantial deviation may significantly affect statistical power and representativeness. The authors should justify the adequacy of this reduced sample size and discuss its implications.

Clarification: Due to strict field eligibility requirements and high on-site rejection rates, the final sample of 200 participants falls short of the projected objective of 384, but it nevertheless offers a solid descriptive baseline with a margin of error of around 6.93%. This size is methodologically appropriate for exploratory cross-sectional analysis at the regional level, and its main implication—a possible decrease in statistical power for more subtle sociodemographic correlations—has been openly added to the study's limitations.

The sampling strategy is insufficiently described. It is unclear how the pharmacies were selected, how many pharmacies participated, and whether the sample can be considered representative of community pharmacy users in the Hail region.

Clarification: A convenient sample strategy was applied across many significant community pharmacies spread across various districts in the Hail region to guarantee broad geographic coverage. Although the convenience approach and final sample size (N=200) restrict real probabilistic representativeness, the use of high-traffic pharmacies in several locations guarantees that the data includes a wide range of local pharmacy consumers.

The manuscript states that the questionnaire was “validated,” but no information is provided regarding validation within the Saudi context or for the current study population. Reporting reliability measures (e.g., Cronbach’s alpha) for the current dataset would strengthen methodological rigor.

Clarification: Thanks. Although the questionnaire developed by Hasan et al was used as source of developing the questionnaire, our questionnaire also contained some questions related to sociodemographic characteristics of the participant, and health status, and it was pretested and reliability measures mentioned in the manuscript under “Study Tool” section represents the Cronbach’s alpha of our questionnaire in context to Saudi Arabia.

The results section is primarily descriptive and lacks deeper interpretation of the findings. Several statistically significant associations are reported but are not adequately contextualized or explained.

Clarification: Thanks. We have explained and interpreted the findings in depth as evident in track-change.

The discussion section largely reiterates the results rather than critically interpreting them. The authors should compare their findings with more international studies and discuss potential cultural, regulatory, or healthcare system factors influencing patient satisfaction.

Clarification: Thanks. We have addressed the comments, and explained in discussion.

Some interpretations in the discussion appear speculative. For example, the explanation regarding uninsured patients relying more on pharmacies is presented without supporting evidence or references.

Clarification: Thanks. We have added two references to support the statement.

The conclusion appears somewhat generalized relative to the limited scope of the study. Given the cross-sectional design and relatively small sample size, stronger caution should be applied when generalizing the findings.

Clarification: Thanks. We have addressed the valuable comments, and the conclusion has been rewritten.

Finally, the manuscript would benefit from clearer reporting following STROBE guidelines for cross-sectional studies, particularly regarding participant recruitment, sampling methods, and statistical analysis.

Clarification: Thanks. We have incorporated the required details in method section that can be seen in track-change.
