# Supplementary material for: Patient satisfaction towards pharmacists’ services in the community pharmacies in the Hail region
Source: PLoS One. 2026 Jul 30;21(7):e0354731. doi: 10.1371/journal.pone.0354731 (PMC13423062; doi:10.1371/journal.pone.0354731)
Supplement: Peer review history

# Peer review: Decision Letter 0

**DOI:** 10.1371/journal.pone.0354731.r001

**Authors:** Yaser Mohammed Al-Worafi

25 Mar 2026

PONE-D-25-61986

Patient satisfaction towards pharmacists’ services in the community pharmacies in the Hail region

PLOS One

Dear Dr.  Ansari,

Thank you for submitting your manuscript to PLOS ONE. After careful consideration, we feel that it has merit but does not fully meet PLOS ONE’s publication criteria as it currently stands. Therefore, we invite you to submit a revised version of the manuscript that addresses the points raised during the review process.

Please submit your revised manuscript by May 09 2026 11:59PM. If you will need more time than this to complete your revisions, please reply to this message or contact the journal office at plosone@plos.org. When you're ready to submit your revision, log on to https://www.editorialmanager.com/pone/ and select the 'Submissions Needing Revision' folder to locate your manuscript file.

Please include the following items when submitting your revised manuscript:

- A letter that responds to each point raised by the academic editor and reviewer(s). You should upload this letter as a separate file labeled 'Response to Reviewers'.
- A marked-up copy of your manuscript that highlights changes made to the original version. You should upload this as a separate file labeled 'Revised Manuscript with Track Changes'.
- An unmarked version of your revised paper without tracked changes. You should upload this as a separate file labeled 'Manuscript'.

If you would like to make changes to your financial disclosure, please include your updated statement in your cover letter. Guidelines for resubmitting your figure files are available below the reviewer comments at the end of this letter.

If applicable, we recommend that you deposit your laboratory protocols in protocols.io to enhance the reproducibility of your results. Protocols.io assigns your protocol its own identifier (DOI) so that it can be cited independently in the future. For instructions see: https://journals.plos.org/plosone/s/submission-guidelines#loc-laboratory-protocols. Additionally, PLOS ONE offers an option for publishing peer-reviewed Lab Protocol articles, which describe protocols hosted on protocols.io. Read more information on sharing protocols at https://plos.org/protocols?utm_medium=editorial-email&utm_source=authorletters&utm_campaign=protocols.

As the corresponding author, your ORCID iD is verified in the submission system and will appear in the published article. PLOS supports the use of ORCID, and we encourage all coauthors to register for an ORCID iD and use it as well. Please encourage your coauthors to verify their ORCID iD within the submission system before final acceptance, as unverified ORCID iDs will not appear in the published article. *Only*  the individual author can complete the verification step; PLOS staff *cannot*  verify ORCID iDs on behalf of authors.

We look forward to receiving your revised manuscript.

Kind regards,

Yaser Mohammed Al-Worafi

Academic Editor

PLOS One

Journal Requirements:

When submitting your revision, we need you to address these additional requirements.

1.Please ensure that your manuscript meets PLOS ONE's style requirements, including those for file naming. The PLOS ONE style templates can be found at

https://journals.plos.org/plosone/s/file?id=wjVg/PLOSOne_formatting_sample_main_body.pdf and

https://journals.plos.org/plosone/s/file?id=ba62/PLOSOne_formatting_sample_title_authors_affiliations.pdf

2. We note that the grant information you provided in the ‘Funding Information’ and ‘Financial Disclosure’ sections do not match.

When you resubmit, please ensure that you provide the correct grant numbers for the awards you received for your study in the ‘Funding Information’ section.

3. Thank you for stating the following financial disclosure:

“This research has been funded by Scientific Research Deanship at University of Ha’il - Saudi Arabia through project number RG-23 038.”

Please state what role the funders took in the study.  If the funders had no role, please state: "The funders had no role in study design, data collection and analysis, decision to publish, or preparation of the manuscript."

If this statement is not correct you must amend it as needed.

Please include this amended Role of Funder statement in your cover letter; we will change the online submission form on your behalf.

4. In the online submission form you indicate that your data is not available for proprietary reasons and have provided a contact point for accessing this data. Please note that your current contact point is a co-author on this manuscript. According to our Data Policy, the contact point must not be an author on the manuscript and must be an institutional contact, ideally not an individual. Please revise your data statement to a non-author institutional point of contact, such as a data access or ethics committee, and send this to us via return email. Please also include contact information for the third party organization, and please include the full citation of where the data can be found.

5. If the reviewer comments include a recommendation to cite specific previously published works, please review and evaluate these publications to determine whether they are relevant and should be cited. There is no requirement to cite these works unless the editor has indicated otherwise.

[Note: HTML markup is below. Please do not edit.]

Reviewer's Responses to Questions

**Comments to the Author**

1. Is the manuscript technically sound, and do the data support the conclusions?

The manuscript must describe a technically sound piece of scientific research with data that supports the conclusions. Experiments must have been conducted rigorously, with appropriate controls, replication, and sample sizes. The conclusions must be drawn appropriately based on the data presented.

Reviewer #1: Yes

Reviewer #2: Yes

Reviewer #3: Partly

**********

2. Has the statistical analysis been performed appropriately and rigorously?

Reviewer #1: Yes

Reviewer #2: Yes

Reviewer #3: Yes

**********

3. Have the authors made all data underlying the findings in their manuscript fully available?

The PLOS Data policy requires authors to make all data underlying the findings described in their manuscript fully available without restriction, with rare exception (please refer to the Data Availability Statement in the manuscript PDF file). The data should be provided as part of the manuscript or its supporting information, or deposited to a public repository. For example, in addition to summary statistics, the data points behind means, medians and variance measures should be available. If there are restrictions on publicly sharing data—e.g. participant privacy or use of data from a third party—those must be specified.

Reviewer #1: Yes

Reviewer #2: Yes

Reviewer #3: Yes

**********

4. Is the manuscript presented in an intelligible fashion and written in standard English?

PLOS ONE does not copyedit accepted manuscripts, so the language in submitted articles must be clear, correct, and unambiguous. Any typographical or grammatical errors should be corrected at revision, so please note any specific errors here.

Reviewer #1: Yes

Reviewer #2: Yes

Reviewer #3: Yes

**********

5. Review Comments to the Author

Please use the space provided to explain your answers to the questions above. You may also include additional comments for the author, including concerns about dual publication, research ethics, or publication ethics. (Please upload your review as an attachment if it exceeds 20,000 characters)

Reviewer #1: My comments are attached within the attached manuscript.

I would recommend you to avail proofreading services so that the manuscript conforms to the academic standards of writing.

Please try to establish the uniqueness in your study so it could add to its significance.

Also report actions you might have taken to address the limitations of the study.

Reviewer #2: 1. How many community pharmacies have been included in the study? From each community pharmacy, how many subjects have been taken in study.

2. More than half or less subjects had high school education in the study as mentioned in the abstract, however, in the conclusion part of the abstract, it is mentioned that mostly well-educated patients availed the community pharmacy services. Is it high school passed patients were considered as well educated in hail region? Similarly, married (60.5%) vs mostly unmarried (percentage is less) percentage patients utilized the pharmacy services. How it can be mostly with unmarried patients if their numbers are lesser than married. Word selection in sentences is not appropriate.

3. Keyword- Please look into keywords mentioned in the abstract, it should be according to standardized MeSH terms.

4. As it is a cross-sectional, face to face survey, why so much drop-outs of the respondents and no responses too. Usually drop-outs are possible in long duration, interventional studies. In cross sectional study, the responses are taken at specific point of time that limits drop-outs. Too much dropouts and low responses will not give the clear outcome of the study. Here in this study, it is almost 50% response only.

5. As this study was conducted through face-to-face survey using cross-sectional design, how the researcher controlled on personal and information biases.

6. About 60% population cannot be indicated as two-third of the subject as mentioned in result section, first paragraph.

7. Under limitation section, what about not achieving the targeted sample in the study and response rate? Are they not considered as limitations?

8. In discussion section, under demographic profile, it is mentioned that most participants were young adults between 18-29 years of age but in Table 1, the categorization of age is given from 23-29 years, at the same time there is no equal categorization of age groups.

9. Table 1 mentioned about 51% of the subjects were not on medication (no medication prescribed), then in that case what type or kind of community pharmacy services they received and why did they go to the community pharmacy?

10. English grammar and phrases need to be rechecked and revised.

Reviewer #3: The manuscript addresses an important topic—patient satisfaction with community pharmacy services in the Hail region. However, the novelty and scientific contribution of the study remain limited. Similar cross-sectional studies assessing patient satisfaction in community pharmacies have already been widely conducted in Saudi Arabia and internationally. The authors should clearly articulate what new knowledge this study adds beyond existing literature.

The introduction is overly descriptive and lacks a clearly defined research gap. While several previous Saudi studies are cited, the authors do not critically compare those findings nor explain how the current study advances the field. The rationale for focusing specifically on the Hail region needs stronger justification.

The sampling procedure raises methodological concerns. Although the authors calculated a required sample size of 384, the final sample included only 200 participants. This substantial deviation may significantly affect statistical power and representativeness. The authors should justify the adequacy of this reduced sample size and discuss its implications.

The sampling strategy is insufficiently described. It is unclear how the pharmacies were selected, how many pharmacies participated, and whether the sample can be considered representative of community pharmacy users in the Hail region.

The manuscript states that the questionnaire was “validated,” but no information is provided regarding validation within the Saudi context or for the current study population. Reporting reliability measures (e.g., Cronbach’s alpha) for the current dataset would strengthen methodological rigor.

The results section is primarily descriptive and lacks deeper interpretation of the findings. Several statistically significant associations are reported but are not adequately contextualized or explained.

The discussion section largely reiterates the results rather than critically interpreting them. The authors should compare their findings with more international studies and discuss potential cultural, regulatory, or healthcare system factors influencing patient satisfaction.

Some interpretations in the discussion appear speculative. For example, the explanation regarding uninsured patients relying more on pharmacies is presented without supporting evidence or references.

The conclusion appears somewhat generalized relative to the limited scope of the study. Given the cross-sectional design and relatively small sample size, stronger caution should be applied when generalizing the findings.

Finally, the manuscript would benefit from clearer reporting following STROBE guidelines for cross-sectional studies, particularly regarding participant recruitment, sampling methods, and statistical analysis.

**********

6. PLOS authors have the option to publish the peer review history of their article (what does this mean?). If published, this will include your full peer review and any attached files.

If you choose “no”, your identity will remain anonymous but your review may still be made public.

**Do you want your identity to be public for this peer review?**  For information about this choice, including consent withdrawal, please see our Privacy Policy.

Reviewer #1: **Yes:**  Dr. Ahlam Sundus

Reviewer #2: No

Reviewer #3: No

**********

[NOTE: If reviewer comments were submitted as an attachment file, they will be attached to this email and accessible via the submission site. Please log into your account, locate the manuscript record, and check for the action link "View Attachments". If this link does not appear, there are no attachment files.]

To ensure your figures meet our technical requirements, please review our figure guidelines: https://journals.plos.org/plosone/s/figures

You may also use PLOS’s free figure tool, NAAS, to help you prepare publication quality figures: https://journals.plos.org/plosone/s/figures#loc-tools-for-figure-preparation.

NAAS will assess whether your figures meet our technical requirements by comparing each figure against our figure specifications.

Attachment Submitted filename: PONE-D-25-61986_reviewer.pdf

# Author comment: Author response to Decision Letter 1

**DOI:** 10.1371/journal.pone.0354731.r002

5 Jul 2026

Response to Journal Requirements

PONE-D-25-61986

Patient satisfaction towards pharmacists’ services in the community pharmacies in the Hail region

Journal Requirements:

When submitting your revision, we need you to address these additional requirements.

1.Please ensure that your manuscript meets PLOS ONE's style requirements, including those for file naming. The PLOS ONE style templates can be found at

https://journals.plos.org/plosone/s/file?id=wjVg/PLOSOne_formatting_sample_main_body.pdf

and https://journals.plos.org/plosone/s/file?id=ba62/PLOSOne_formatting_sample_title_authors_affiliations.pdf

Clarification: Thanks. We have checked thoroughly, and we hope the manuscript comply with the journal’s requirements.

2. We note that the grant information you provided in the ‘Funding Information’ and ‘Financial Disclosure’ sections do not match.

When you resubmit, please ensure that you provide the correct grant numbers for the awards you received for your study in the ‘Funding Information’ section.

Clarification: Thanks. We have checked, and assure that the grant number is correct.

3. Thank you for stating the following financial disclosure:

“This research has been funded by Scientific Research Deanship at University of Ha’il - Saudi Arabia through project number RG-23 038.”

Please state what role the funders took in the study. If the funders had no role, please state: "The funders had no role in study design, data collection and analysis, decision to publish, or preparation of the manuscript."

If this statement is not correct you must amend it as needed.

Please include this amended Role of Funder statement in your cover letter; we will change the online submission form on your behalf.

Clarification: Thanks. We have amended in the cover letter.

4. In the online submission form you indicate that your data is not available for proprietary reasons and have provided a contact point for accessing this data. Please note that your current contact point is a co-author on this manuscript. According to our Data Policy, the contact point must not be an author on the manuscript and must be an institutional contact, ideally not an individual. Please revise your data statement to a non-author institutional point of contact, such as a data access or ethics committee, and send this to us via return email. Please also include contact information for the third party organization, and please include the full citation of where the data can be found.

Clarification: Thanks. The data is made available

5. If the reviewer comments include a recommendation to cite specific previously published works, please review and evaluate these publications to determine whether they are relevant and should be cited. There is no requirement to cite these works unless the editor has indicated otherwise.

Clarification: Not applicable

Attachment Submitted filename: Response to Reviewer 3.docx

# Editor report: Decision Letter 1

**DOI:** 10.1371/journal.pone.0354731.r003

**Authors:** Yaser Mohammed Al-Worafi

13 Jul 2026

Patient satisfaction towards pharmacists’ services in the community pharmacies in the Hail region

PONE-D-25-61986R1

Dear Dr.Ansari,

We’re pleased to inform you that your manuscript has been judged scientifically suitable for publication and will be formally accepted for publication once it meets all outstanding technical requirements.

Within one week, you’ll receive an e-mail detailing the required amendments. When these have been addressed, you’ll receive a formal acceptance letter and your manuscript will be scheduled for publication.

An invoice will be generated when your article is formally accepted. Please note, if your institution has a publishing partnership with PLOS and your article meets the relevant criteria, all or part of your publication costs will be covered. Please make sure your user information is up-to-date by logging into Editorial Manager at Editorial Manager® and clicking the ‘Update My Information' link at the top of the page. For questions related to billing, please contact billing support.

If your institution or institutions have a press office, please notify them about your upcoming paper to help maximize its impact. If they’ll be preparing press materials, please inform our press team as soon as possible -- no later than 48 hours after receiving the formal acceptance. Your manuscript will remain under strict press embargo until 2 pm Eastern Time on the date of publication. For more information, please contact onepress@plos.org.

Kind regards,

Yaser Mohammed Al-Worafi

Academic Editor

PLOS One

Additional Editor Comments (optional):

Reviewers' comments:

# Editor report: Acceptance letter

**DOI:** 10.1371/journal.pone.0354731.r004

**Authors:** Yaser Mohammed Al-Worafi

PONE-D-25-61986R1

PLOS One

Dear Dr. Ansari,

I'm pleased to inform you that your manuscript has been deemed suitable for publication in PLOS One. Congratulations! Your manuscript is now being handed over to our production team.

At this stage, our production department will prepare your paper for publication. This includes ensuring the following:

* All references, tables, and figures are properly cited

* All relevant supporting information is included in the manuscript submission,

* There are no issues that prevent the paper from being properly typeset

You will receive further instructions from the production team, including instructions on how to review your proof when it is ready. Please keep in mind that we are working through a large volume of accepted articles, so please give us a few days to review your paper and let you know the next and final steps.

Lastly, if your institution or institutions have a press office, please let them know about your upcoming paper now to help maximize its impact. If they'll be preparing press materials, please inform our press team within the next 48 hours. Your manuscript will remain under strict press embargo until 2 pm Eastern Time on the date of publication. For more information, please contact onepress@plos.org.

You will receive an invoice from PLOS for your publication fee after your manuscript has reached the completed accept phase. If you receive an email requesting payment before acceptance or for any other service, this may be a phishing scheme. Learn how to identify phishing emails and protect your accounts at https://explore.plos.org/phishing.

If we can help with anything else, please email us at customercare@plos.org.

Thank you for submitting your work to PLOS ONE and supporting open access.

Kind regards,

PLOS ONE Editorial Office Staff

on behalf of

Professor Yaser Mohammed Al-Worafi

Academic Editor

PLOS One
